# Supplementary material for: IgE and Eosinophilia in Newly Arrived Refugees in Denmark: A Cross-Sectional Study of Prevalence and Clinical Management in Primary Care
Source: Int J Environ Res Public Health. 2025 Jan 28;22(2):180. doi: 10.3390/ijerph22020180 (PMC11855504; doi:10.3390/ijerph22020180)
Supplement: Supplementary file 1 [file ijerph-22-00180-s001.zip › ijerph-3413683-supplementary.pdf]

Supplementary

Table S1. Logistic regression for elevated IgE separated by country.

Country of origin	Odds for elevated IgE			
	Unadjusted		Adjusted*	
	OR [95%CI]	P-value	OR [95%CI]	P-value
**Syria**	0.24 [0.16;0.36]	<0.001	0.24 [0.16;0.36]	<0.001
**Lebanon**	1.44 [0.30;7.00]	0.653	1.61 [0.32;7.90]	0.560
**Iran**	2.75 [1.67;4.51]	<0.001	2.76 [1.67;4.54]	<0.001
**Iraq**	1.11 [0.24;5.22]	0.890	1.14 [0.24;5.35]	0.870
**Afghanistan**	3.22 [1.71;6.05]	<0.001	3.02 [1.60;5.71]	0.001
**Somalia**	2.22 [1.07;4.61]	0.033	2.21 [1.06;4.62]	0.035
**Eritrea**	2.52 [1.39;4.56]	0.002	2.58 [1.41;4.70]	0.002
**Ethiopia**	2.54 [0.63;10.28]	0.192	2.75 [0.67;11.21]	0.195
**Congo**	Not enough data			
**Unknown country, Western Asia region**	Not enough data			
**Other****	Not enough data			

*Adjusted for age and sex. **Countries with less than five participants: Pakistan, Libya, Jordan, Kuwait, Zambia, Morocco, Central African Republic, Russia, Columbia.
